# Effect of mining activities on the rhizosphere soil bacteria of seven plants in the iron ore area

**DOI:** 10.3389/fmicb.2025.1728759

**Published:** 2025-12-17

**Authors:** Xiyue Yan, Xingting Huang, Bingliang Liu, Huimei Pan, Qiang Li

**Affiliations:** 1 Key Laboratory of Coarse Cereal Processing, Ministry of Agriculture and Rural Affairs, Sichuan Engineering and Technology Research Center of Coarse Cereal Industrialization, School of Food and Biological Engineering, Chengdu University, Chengdu, China; 2 Solar Energy Utilization Technology Integration Engineering Laboratory of Sichuan Province, Panzhihua University, Panzhihua, China

**Keywords:** actinomycetes, bacterial diversity, bioremediation, iron ore area, rhizosphere soil

## Abstract

**Introduction:**

Mining-induced changes in soil composition, coupled with the persistent challenge of heavy metal pollution, drive structural shifts in soil bacterial communities.

**Methods:**

This study employed ICP-OES and other methods to analyze soil physicochemical properties and performed 16S rRNA sequencing of rhizosphere bacteria to investigate the impact of iron ore mining on the diversity and composition of rhizosphere bacteria in the western Yunnan-Guizhou Plateau.

**Results:**

The results indicated that the soil nutrient and heavy metal concentrations underwent notable changes due to mining activities. Rhizosphere soil was significantly enriched in Fe and Cu (*p* < 0.05), but was deficient in alkaline nitrogen (AN) and available phosphorus (AP). Microbial α-diversity was positively correlated with total nitrogen (TN) and organic carbon (OC) but negatively correlated with available potassium (AK) and heavy metal content. Polygonum plebeium R.Br. significantly enriched multidrug-resistant Pseudarthrobacter, while the rhizosphere of Casuarina equisetifolia L. was dominated by the oligotrophic bacterium RB41.

**Discussion:**

This study reveals critical plant-microbe interactions in a mining-disturbed ecosystem and provides a scientific basis for developing microbial indicators and microbe-oriented restoration strategies.

## 1 Introduction

The rapid development of the global economy drives an irreversible demand for mineral resources (Wong et al., 2024). However, large-scale mining operations inevitably lead to severe environmental degradation. This includes widespread vegetation destruction, as evidenced in iron ore areas where mining leads to dramatic loss of plant cover and ecosystem stability (Pang et al., 2018; Song et al., 2024). In addition, mining causes profound soil disturbance and alters fundamental soil properties; for instance, studies at iron mine tailings dams reveal that soil organic carbon pools can remain severely depleted even years after rehabilitation efforts (Nie et al., 2018; Demin et al., 2015). The release of heavy metals from mine waste further exacerbates these issues, causing contamination that not only inhibits plant growth (Li Y. et al., 2025) but also disrupts entire ecosystems and threatens human health (Entwistle et al., 2019; Hui, 2025; Li Y. et al., 2025; Macklin et al., 2023; Zhanwei et al., 2015; Zhang et al., 2024). Therefore, remediating heavy metal-contaminated soils represents a critical and urgent global challenge.

Soil microorganisms play a crucial role in terrestrial ecosystem functions. They are involved in important biogeochemical processes like carbon and nitrogen cycling (Adamczyk et al., 2025; Hu et al., 2024; Shen et al., 2022), organic matter mineralization (Su et al., 2023), and regulating plant growth and stress resistance through various pathways such as nitrogen fixation, phosphorus solubilization, potassium solubilization, organic matter decomposition, and hormone secretion (Bhat et al., 2022; Cao et al., 2022; Li J. et al., 2023; Li et al., 2021; Sharma et al., 2025; Vaghela and Gohel, 2025; Wang et al., 2025). In contaminated soil environments, the plant rhizosphere plays a key role as a significant “microbial hotspot”(Tang et al., 2025; Wang et al., 2024) connecting plants and soil to aid plants in resisting heavy metal toxicity through mechanisms such as extracellular precipitation and organic acid secretion. This highlights its importance for ecosystem restoration (Li X. et al., 2023; Jie et al., 2023). However, the assembly of this microbial community is simultaneously influenced by environmental selection driven by soil physicochemical properties such as pH, nutrients, and heavy metals (Bai et al., 2023; Guo et al., 2024). Extensive research confirms that mining activities significantly reshape soil microbial communities and reduce their diversity, prompting growing academic interest in rhizosphere microbial behavior under specific mining conditions. For instance, in lead-zinc tailings, rhizosphere microbes (such as Proteobacteria and Actinobacteria) maintain ecosystem stability by highly expressing resistance genes like zntA and pbrB (Zhu et al., 2025); Tomentella has been reported as a dominant symbiotic mycorrhizal fungus in *Pinus massoniana* root zones (Huang et al., 2012); while Pseudomonas exhibits high tolerance to multiple heavy metals including Cd, Cu, Cr, Zn, and Ni in agricultural fields (Guo et al., 2023) and as hydrocarbon-degrading bacteria (Disi et al., 2022). Furthermore, studies by Zhang et al. (2024) and Sun et al. (2018) further revealed the core microbial communities (e.g., *Tomentella* and *Cladosporium*) in the rhizosphere of *Miscanthus sinensis*, as well as the patterns of specific responses to V and Cr by microbial communities in different crop rhizospheres. These findings collectively indicate the strong adaptability and potential metal cycling functions of specific microbial groups in heavy metal-polluted environments.

Various plants can influence distinct rhizosphere microbial communities through mechanisms like root exudates. However, the specific responses and mechanisms of multiple plant rhizosphere microbiomes under mining disturbance are not well understood. There is a lack of systematic comparative studies on rhizosphere bacterial communities of multiple native plants in typical iron ore mining habitats. Additionally, there is a dearth of quantitative analyses on how soil physicochemical factors (e.g., pH, organic matter, heavy metals) collectively impact *α* diversity and core bacterial genus composition in different plant rhizosphere bacteria. To address these gaps, this study examined seven typical native plant species from an iron ore mining area in the western Yunnan-Guizhou Plateau. Rhizosphere soil samples were subjected to high-throughput sequencing and soil physicochemical analyses to investigate: (1) Variations in bacterial community structure and diversity across plant rhizosphere environments; and (2) The influential roles of key environmental factors in community assembly. This research offers theoretical insights into plant-microbe interactions in mining-disturbed environments and establishes a scientific basis for developing microbial formulations and vegetation strategies for ecological restoration in iron ore mining areas.

## 2 Materials and methods

### 2.1 Rhizosphere soil collection

In an iron ore area in the western Yunnan-Guizhou Plateau, seven representative plant species were collected, and the coverage rate of these seven plant species accounted for approximately 70%. The plants of the rhizosphere soil sampled were *Polygonum plebeium R.Br*., *Tournefortia sibirica* L., *Casuarina equisetifolia* L., *Pteris vittata* L., *Dodonaea viscosa Jacquem.*, *Dryopteris coreano-montana* Nakai and *Alhagi camelorum* Fisch. The sampling location was the ilmenite ore area near Wuding County, Chuxiong Yi Autonomous Prefecture, Yunnan Province (latitude and longitude: 102°24′14.389″E, 25°31′49.058”N). The *Polygonum plebeium R.Br*., *Tournefortia sibirica* L., *Casuarina equisetifolia* L., *Pteris vittata* L., *Dodonaea viscosa Jacquem.*, *Dryopteris coreano-montana* Nakai and *Alhagi camelorum* Fisch., from the ilmenite area were marked as Ppl, Tsi, Ceq, Pvi, Dvi, Dco and Aca. The bulk soil without plantings collected from a non-ilmenite area in a similar environment was used as the total control sample and was named KB. Three quadrats, each with an area of 2 m^2^, were randomly established in the mining area. The rhizosphere soil samples were collected at multiple points in the same quadrant and evenly mixed to form one sample. Each sample had three biological replicates. Plant root systems and soil samples were collected at a depth of 0–20 cm. After shaking off the loose soil from the roots, the root soil of 0–5 mm was collected with a sterile brush and then sieved to remove impurities. A single sample was placed in sterile sampling bags and stored at −80 °C for soil microbial DNA extraction and high-throughput sequencing. The second sample was utilized for analyzing soil physicochemical properties and detecting heavy metals.

### 2.2 DNA extraction, PCR amplification, and detection

The soil genomic DNA were obtained utilizing the standard protocol following the CTAB extraction. To evaluate both the purity and concentration of the isolated genetic material, we ran agarose gel electrophoresis. Following this analysis, the DNA was transferred to a centrifuge tube and then brought down to a working concentration of 1 ng/μL by adding sterile water.

Using the diluted genomic DNA as a template, specific primers with barcode (338F (5’-ACTCCTACGGGAGGCAGCAG-3′) and 806R (5’-GGACTACHVGGGTWTCTAAT-3′)) were selected according to the amplified region, New England Biolabs Inc. Phusion® High-Fidelity PCR Master Mix with GC Buffer and high-efficiency high-fidelity enzymes were used for PCR to ensure efficient and accurate amplification.

After running the PCR products on a 2% agarose gel to visualize them, we combined equal volumes of each sample according to their concentration levels. The mixture was then thoroughly shaken before being run through another 2% agarose gel electrophoresis for visualization. Finally, we used a commercially available gel recovery kit to extract and purify the DNA fragments.

### 2.3 Library preparation, sequencing, and raw data processing

The NEB Next® Ultra™ II DNA Library Prep Kit was used for library construction. The library was quantified using Qubit and qPCR before being sequenced on the NovaSeq6000. Once the library was qualified, the data of each sample were divided from the off-loading data based on the barcode sequence and PCR amplification primer sequences. The barcode and primer sequences were then truncated and used in FLASH (V1.2.11)^1^ (Magoc and Salzberg, 2011). The software stitched together the sample reads to generate initial Raw Tags. Next, Fastp stepped in to put these Raw Tags through their paces, ensuring only the highest quality Clean Tags made the cut. In the final stage, U search was called upon to match the Clean Tags against the database, sniffing out and eliminating any chimeric sequences (Haas et al., 2011). This rigorous process culminated in the production of Effective Tags, representing the cream of the crop in terms of data reliability.

### 2.4 Microbial taxonomic profiling

The Effective Tags underwent quality control, noise reduction, and filtering out of sequences with an abundance of less than 5. The sequences generated were ASVs or signature sequences, which corresponded to the OTU representative sequences and were classified using QIIME2. The ASVs were obtained using the sklearn algorithm (Bokulich et al., 2018; Bolyen et al., 2019). The ASVs obtained were cross-referenced with the database to determine the species associated with each ASV. Based on the ASV annotation results and the characteristic table of each sample, the species abundance table at the kingdom, phylum, class, order, family, genus, and species level was obtained. Based on the species annotation results, the top 10 species with the highest abundance at each taxonomic level (Phylum, Class, Order, Family, Genus) were selected to generate a columnar cumulative chart of relative abundance for each sample or grouping. This allows for a visual representation of the species annotation results. Species with greater relative abundance and their proportions at various taxonomic levels were incorporated.

### 2.5 *α*-diversity and *β*-diversity analysis

The QIIME2 software calculated the Observed_otus, Shannon, Simpson, Chao1, Goods_coverage, Dominance, and Pielou_e indices, and drew the dilution curve and species accumulation boxplot. The Unifrac distance was calculated using QIIME2 software, and R software was used to draw the PCA, PCoA, and NMDS dimension reduction diagrams to analyze the inter-group difference in alpha diversity in case grouping exists. The ade4 and ggplot2 packages in R software are essential for performing PCA and PCoA. Following this, the adonis and anosim functions in QIIME2 software were employed to assess the significance of variations in community structure among groups. Species analysis of significant differences among groups was conducted using LEfSe or R software. For LEfSe analysis, the LDA score threshold was set to 4 by default. In MetaStat analysis, R software was used to conduct the difference test between the two comparison groups at the six classification levels of phylum, class, order, family, genus and species and obtain the *p* value. Species with a *p* value less than 0.05 were considered significant between groups. The *t*-test was also conducted using R software to determine significant differences among species at each taxonomic level.

### 2.6 Function prediction of rhizosphere bacteria

PICRUSt2 is a bioinformatics tool that predicts metagenome function using marker genes like 16S rRNA. It predicts functions based on databases like KEGG, COG, PFAM, TIGRFAM, EC, and KO from 16S sequencing data.

### 2.7 Tests of soil physicochemical properties

Soil pH was measured through potentiometric titration in a 1:2.5 (w/v) soil-water mixture. Soil organic matter (SOM) was determined using the Walkley-Black wet oxidation technique. Total nitrogen (TN) and alkali-hydrolyzable nitrogen (AN) were analyzed using the Kjeldahl method and alkali-hydrolyzable diffusion method, respectively. Soil available phosphorus (AP) was assessed using the Olsen method, which involves a 0.5 M sodium bicarbonate (pH 8.5) extraction and molybdenum antimony antispectrophotometric analysis. Soil available potassium (AK) was determined by the FAAS method, which involves a 1 M ammonium acetate (pH 7.0) extraction and flame atomic absorption spectroscopy.

The soil samples underwent pretreatment for heavy metal (Cr, Zn, Pb, As) and total element (P, K) analysis by utilizing a mixed acid system of nitric acid-hydrochloric acid-hydrofluoric acid with a microwave digestion system. Post digestion, the concentrations of all elements were determined through an inductively coupled plasma optical emission spectrometer (ICP-OES). To ensure accuracy due to its lower detection limit, Arsenic (As) was measured using an inductively coupled plasma mass spectrometer (ICP-MS).

### 2.8 Data analysis

All data from biological replicates (*n* = 3) are presented as mean ± standard deviation. Initial assessments of diversity indices and soil physicochemical properties were conducted using Excel 2019 software. A two-way ANOVA was employed to evaluate interactions between plant species and soil properties, while Spearman’s correlation analysis using SPSS 23.0 software examined associations between environmental factors and microbial diversity. The significance of differences between groups was determined with a *t*-test for two groups or Tukey’s test for more than two groups. Differences between means were considered statistically significant at the *p* = 0.05 level.

## 3 Results and analysis

### 3.1 Basic properties of soil in the mining area

Soil samples from seven plant species in a mining area were analyzed for pH, total potassium, total nitrogen, and concentrations of Fe, Ti, Cu, Zn, and Pb. The pH of the soil was found to range from 6.91 to 8.04, indicating neutral conditions (Table 1). Iron pollution levels in the rhizosphere of all seven plants were higher than the control group, with values ranging from 77.92 to 142.27 mg/kg (*p* < 0.05). Zinc contents in the rhizosphere of all seven plants were all lower than the control group (109.33 ± 2.08 mg/kg) (*p* < 0.05). Copper contamination values were higher in six out of seven plant groups compared to the control group (112.33 ± 3.21 mg/kg) (*p* < 0.05).

**Table 1:** Basic chemical properties of the rhizosphere soil of the seven plants and the control.

Element	Ppl	Tsi	Aca	Ceq	Dco	Pvi	Dvi	KB
pH	6.94 ± 0.03f	7.21 ± 0.04d	7.22 ± 0.05d	8.02 ± 0.02a	7.40 ± 0.06c	7.02 ± 0.04e	7.77 ± 0.01b	7.15 ± 0.01d
TP	1.73 ± 0.02c	1.83 ± 0.01b	1.38 ± 0.01e	2.12 ± 0.01a	1.65 ± 0.03d	0.92 ± 0.02h	1.34 ± 0.03f	1.04 ± 0.02g
TN	0.29 ± 0.01 g	0.45 ± 0.01e	0.37 ± 0.01f	2.26 ± 0.03a	2.18 ± 0.01b	0.76 ± 0.00d	2.24 ± 0.04a	1.89 ± 0.02c
TK	14.20 ± 0.20f	14.3 ± 0.20f	15.13 ± 0.06e	20.63 ± 0.25c	22.87 ± 0.21a	21.60 ± 0.17b	19.27 ± 0.21d	15.10 ± 0.20e
AP	5.8 ± 0.69d	4.93 ± 0.42d	3.83 ± 0.31e	9.13 ± 0.65c	5.43 ± 0.40d	2.83 ± 0.32e	15.33 ± 0.99b	21.47 ± 0.87a
AN	14.00 ± 1.00g	18.00 ± 1.00f	12.67 ± 1.53 g	157.0 ± 1.00c	152.0 ± 2.00d	44.33 ± 0.58e	165.0 ± 2.65b	218.0 ± 1.00a
OC	2.30 ± 0.08d	2.65 ± 0.08d	1.95 ± 0.04d	27.08 ± 0.23b	26.96 ± 0.87b	4.52 ± 0.17c	26.28 ± 0.85b	28.16 ± 0.20a
AK	281.67 ± 2.52b	367.0 ± 4.36a	286.33 ± 3.51b	251.67 ± 3.21c	187.67 ± 1.53e	238.33 ± 4.51d	281.0 ± 5.29b	144.67 ± 3.06f
Fe	94.03 ± 0.50b	91.80 ± 4.53bc	85.00 ± 4.00cd	135.37 ± 6.90a	81.93 ± 3.11d	81.23 ± 3.31d	95.00 ± 4.92b	73.47 ± 2.21e
Zn	89.67 ± 3.51c	96.67 ± 4.51b	83 ± 4.00d	81.67 ± 4.16d	48.67 ± 1.53f	30.33 ± 0.58g	65.33 ± 3.21e	109.33 ± 2.08a
Cu	130.00 ± 6.08c	137.67 ± 4.16c	152.67 ± 4.16b	319.0 ± 9.54a	105.67 ± 4.16d	48.33 ± 1.15e	154.33 ± 7.09b	112.33 ± 3.21d
Ti	14.57 ± 0.59a	13.63 ± 0.64b	12.00 ± 0.20c	4.23 ± 0.06f	4.80 ± 0.17f	7.00 ± 0.26d	5.60 ± 0.20e	5.83 ± 0.06e
Pb	39.13 ± 1.24e	37.33 ± 1.71e	37.83 ± 1.53e	102.67 ± 3.89a	54.30 ± 1.31d	37.30 ± 1.47e	74.53 ± 3.42b	66.70 ± 2.82c

The letters “a–h” denote the results of significance analysis based on Tukey’s HSD test. Units of measurement for pH, TP, TN, TK, AP, AN, OC, and AK are (g/kg dw), while the units for Fe, Zn, Cu, Ti, and Pb are (mg/kg dw).

The concentrations of macronutrients and key soil properties, essential for plant growth and soil health, varied significantly among the rhizosphere soils of the seven plants and the bulk soil (KB) (Table 1). Regarding nitrogen components, the total nitrogen (TN) content was lower in the rhizosphere of Ppl, Aca, Tsi, and Pvi compared to KB, while Ceq and Dvi showed no significant difference. Similarly, the levels of alkaline nitrogen (AN) and organic carbon (OC) in all seven plant rhizospheres were significantly lower than in KB (*p* < 0.05). For phosphorus components, the total phosphorus (TP) content across the eight soils followed the order: Pvi > KB > Dvi > Aca > Dco > Ppl > Tsi > Ceq, with significant differences (*p* < 0.05). Conversely, available phosphorus (AP) content in all plant rhizospheres was significantly lower than in KB. In the case of potassium, the total potassium (TK) content ranked as Dco > Pvi > Ceq > Dvi > Aca > KB > Tsi > Ppl, with no significant difference between Ppl and Tsi or between KB and Aca. In contrast to TK, the available potassium (AK) content in all plant rhizospheres was significantly higher than in KB. In summary, the measured macronutrients and properties exhibited distinct and significant variations between the plant rhizospheres and the control soil (*p* < 0.05).

### 3.2 ASV analysis of the bacterial communities in the rhizosphere soil

High-throughput sequencing was performed on eight groups of soil samples, resulting in a total of 21,641 ASVs after quality control steps. The sequencing depth was determined to be sufficient, as indicated by the gentle plateauing of dilution curves for all samples, which ensures a comprehensive characterization of the bacterial community composition.

**Figure 1:**
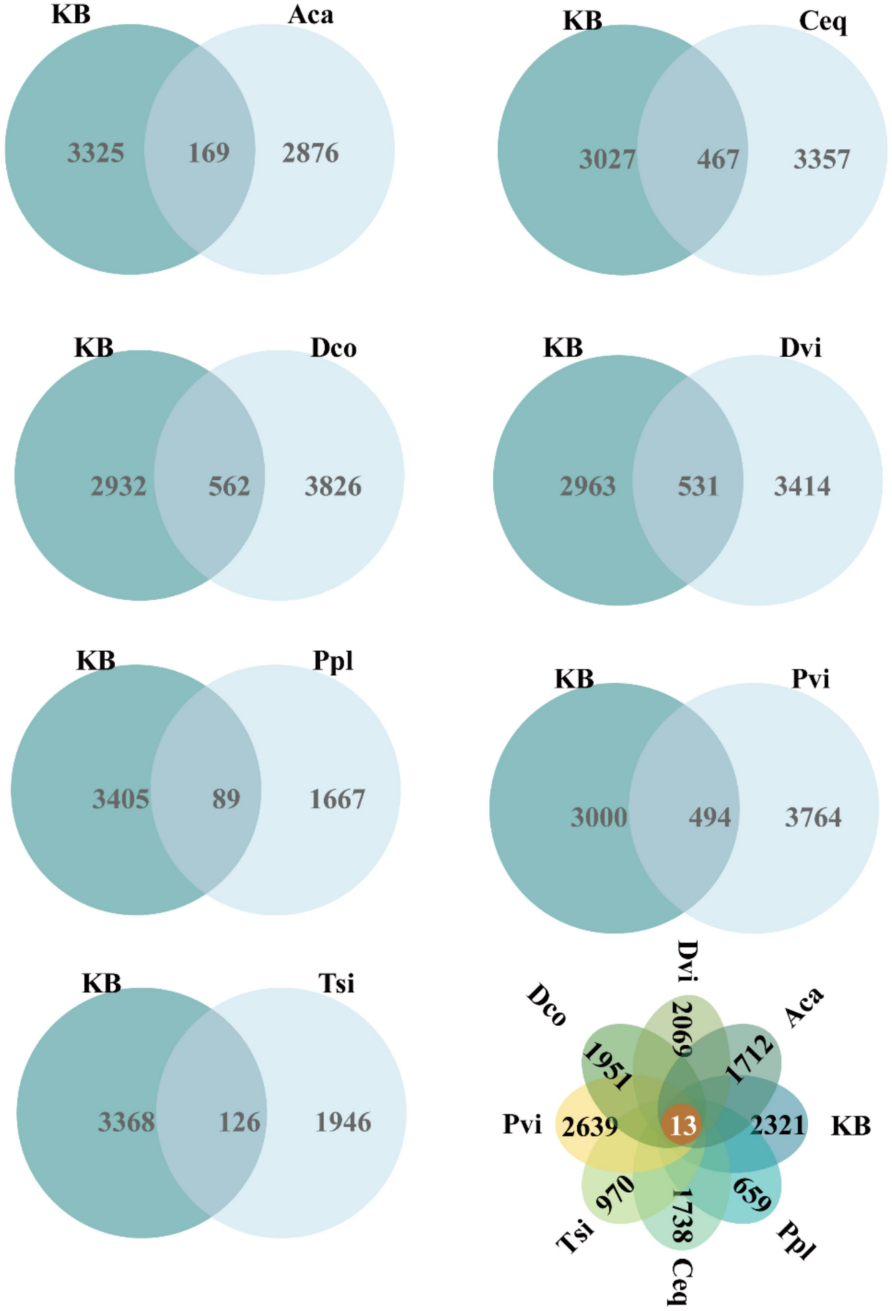
Shared and unique ASV analysis among different samples.

Taxonomic annotation of the ASVs revealed a diverse bacterial community across the soil samples. A total of 50 phyla, 142 classes, 334 orders, 516 families, 1,093 genera, and 1,687 species were identified. As shown in Figure 1, only 13 ASVs were shared among all samples. The number of unique ASVs in each sample followed the order: Pvi > KB > Dvi > Dco > Ceq > Aca > Tsi > Ppl. Specifically, the Pvi rhizosphere harbored the highest richness (2,639 unique ASVs), while the Ppl rhizosphere contained the lowest (659 unique ASVs) (Figure 1).

### 3.3 Bacterial composition in the rhizosphere soil

**Figure 2:**
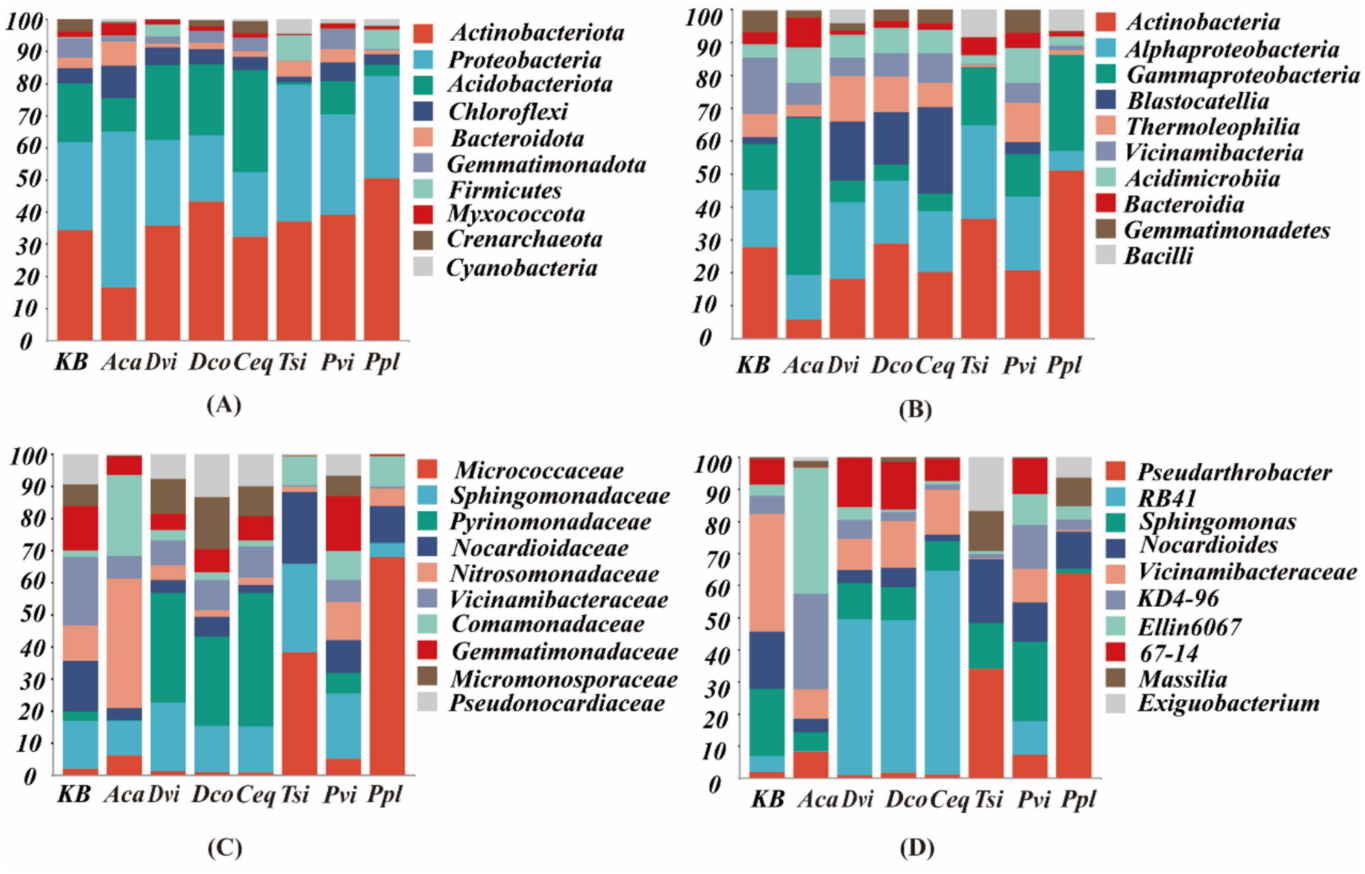
Relative abundance (top 10) of taxa at the **(A)** phylum, **(B)** order, **(C)** family, and **(D)** genus levels of different samples.

The species composition and relative abundance of the Top10 at the phylum, class, family, and genus levels of the rhizosphere soil bacterial communities are shown in Figure 2. A total of 50 bacterial phyla were identified in all soil samples, ranging from Figure 2A. It can be seen from Figure 2A, that among the seven samples from the iron ore area, the main bacterial taxa at the phylum level were Proteobacteria, Actinobacteriota, Acidobacteriota, Chloroflexi, Gemmatimonadota, Bacteroidota, Firmicutes, Cyanobacteria, Myxococcota, and Crenarchaeota, accounting for 95% of all bacterial abundance. Among them, Actinobacteriota had the highest richness, accounting for 32.78% of all bacteria. Compared with the control group (KB), except for Aca and Ceq, the abundance of the remaining five Actinobacteriota species all increased. In particular, Ppl increased by 16.11% compared to KB, and Aca decreased by 17.84% compared to KB. At the class level, as shown in Figure 2B, Actinobacteria, Alphaproteobacteria, and Gammaproteobacteria were the top three bacteria, accounting for 22.37, 15.72 and 13.99%, respectively. In the Actinobacteria taxa, compared to the control group KB, the relative abundance of Aca decreased by 22.02%, and the relative abundance of Ppl increased by 23.23%. In the Alphaproteobacteria family, the relative abundances of Ppl and Aca decreased by 11.3 and 3.76%, respectively. In Gammaproteobacteria, the relative abundance of Aca and Ppl increased by 28.59 and 15.07%, respectively. At the genus level, as shown in Figure 2D, the dominant genus in Ppl was *Pseudarthrobacter*, and its relative abundance increased by 61.99% compared to that in the control group KB, while the relative abundance of Vicinamibacteraceae decreased by 36.07%. The dominant genus of Ceq, Dco and Dvi was all *RB41*, which was 59.47, 42.52 and 43.43% higher than that in the control group, respectively. The dominant genera in Aca were *Ellin6067* and *KD4-96*, which increased by 36.01 and 23.99% respectively, compared to the control group KB.

### 3.4 Bacterial diversity in the rhizosphere soil

**Figure 3:**
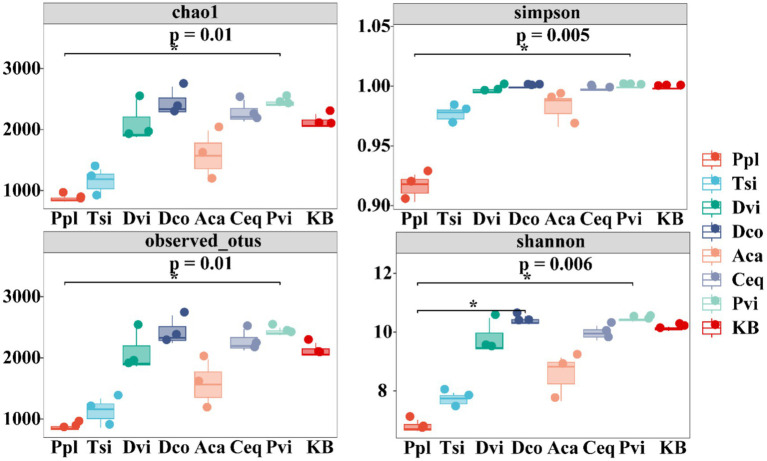
Box plot of alpha diversity index of different samples.

The alpha diversity of rhizosphere bacterial communities across the seven plants was assessed using multiple indices (Figure 3). The richness of the communities, represented by the Chao1 and Observed_otus indices, was highest in Pvi and lowest in Ppl. In contrast, the diversity and evenness of the communities, reflected by the Shannon and Simpson indices, showed a similar pattern, with Pvi exhibiting the highest Shannon index (10.42) and Ppl the lowest (6.32).

**Figure 4:**
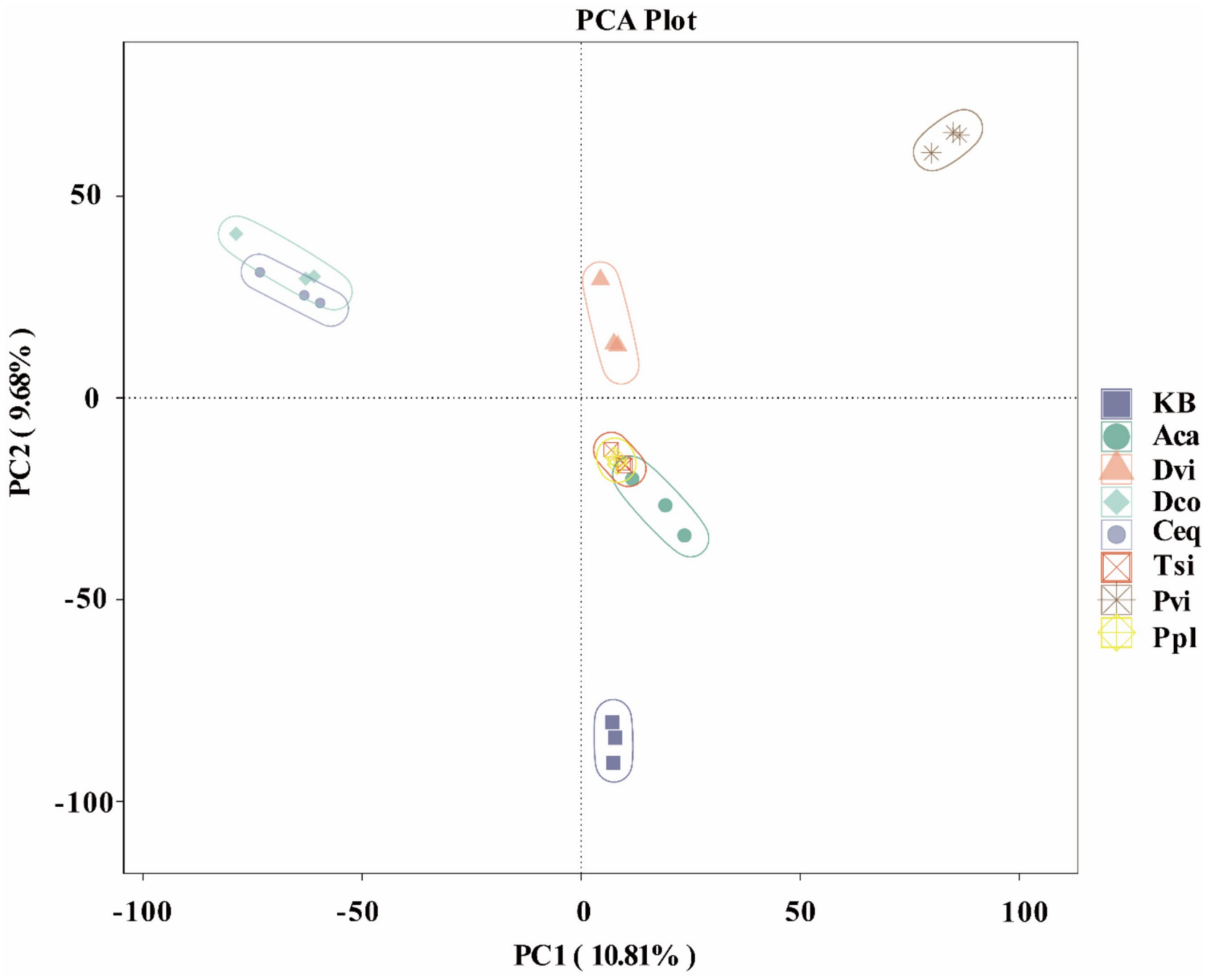
Principal coordinate analysis (PCoA) between different samples based on the weighted Unifrac distance.

Beta diversity was utilized at the ASV level to examine the variations in bacterial community structure among the rhizosphere soils of the seven plants and the blank control group. There are variations in community structure observed among the different samples, as illustrated in Figure 4. The PC1 axis and PC2 axis can reveal 10.81 and 9.68% of the results, respectively. Ppl and Tsi almost completely overlap, Ceq and Dco have a large overlap, and Aca partially overlaps with Ppl and Tsi, but is far from the other groups. This indicates that the overlapping groups share more common bacterial communities.

### 3.5 Function prediction of rhizosphere soil bacteria

**Figure 5:**
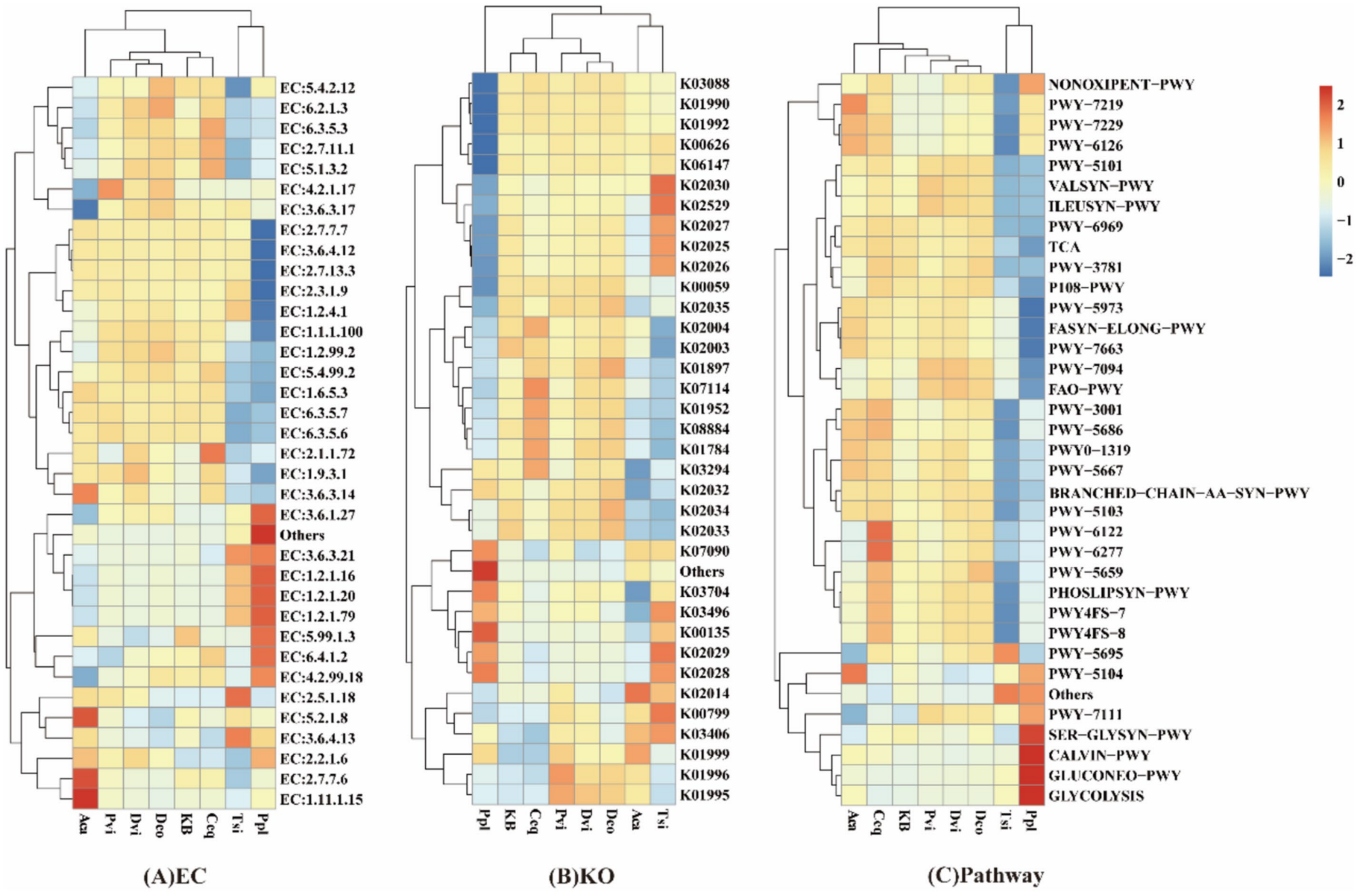
Heatmap of function prediction in rhizosphere soils with different plants. PICRUSt2 function prediction: **(A)** EC, **(B)** KO, **(C)** Pathway.

The prediction of rhizosphere soil bacteria function was carried out using PICRUSt2. The top 35 functions ranked by abundance and their abundance information in each sample were selected from the EC, KO, Pathway, and TIGEs databases. A heat map was generated and clustered at various functional levels (Figure 5).

In the EC database, the relative abundances of genes encoding Peroxiredoxin (EC: 1.11.1.15), DNA-directed RNA polymerase (EC: 2.7.7.6), and Peptidylprolyl isomerase (EC: 5.2.1.8) were significantly higher in the Aca group compared with the control group (*p* < 0.05), while the abundance of Monosaccharide-transporting ATPase (EC: 3.6.3.17) was significantly lower (*p* < 0.05). The abundance of Site-specific DNA-methyltransferase (adenine-specific) (EC: 2.1.1.72) was significantly increased in the Ceq group (*p* < 0.05). The abundances of Glutathione transferase (EC: 2.5.1.18) and RNA helicase (EC: 3.6.4.13) were significantly elevated in the Tsi group (*p* < 0.05). Among all groups, the Ppl group showed the most pronounced changes in predicted gene abundances. For instance, the abundances of genes encoding Undecaprenyl-diphosphate phosphatase (EC: 3.6.1.27), Succinate-semialdehyde dehydrogenase (NAD(P)(+)) (EC: 1.2.1.16), Glutarate-semialdehyde dehydrogenase (EC: 1.2.1.20), Succinate-semialdehyde dehydrogenase (NADP(+)) (EC: 1.2.1.79), DNA topoisomerase (ATP-hydrolyzing) (EC: 5.99.1.3), Acetyl-CoA carboxylase (EC:6.4.1.2), and DNA-(apurinic or apyrimidinic site) lyase (EC:4.2.99.18) were significantly increased. Conversely, the abundances of genes for DNA-directed DNA polymerase (EC: 2.7.7.7), DNA helicase (EC: 3.6.4.12), Histidine kinase (EC: 2.7.13.3), Acetyl-CoA C-acetyltransferase (EC: 2.3.1.9), and Pyruvate dehydrogenase (acetyl-transferring) (EC: 1.2.4.1) were significantly decreased (*p* < 0.05).

In the KO database, the predicted abundances of several genes in the Ppl group were significantly altered. The abundances of rpoE (RNA polymerase sigma-70 factor, ECF subfamily, K03088), *ABC-2.A* (ABC-2 type transport system ATP-binding protein, K01990), *ABC-2.P* (ABC-2 type transport system permease protein, K01992), atoB (acetyl-CoA C-acetyltransferase [EC: 2.3.1.9], K00626), and ABCB-BAC (ATP-binding cassette, subfamily B, bacterial, K06147) were significantly decreased. In the Aca group, the abundance of TC.FEV.OM (iron complex outermembrane receptor protein, K02014) was significantly increased, while TC.APA (basic amino acid/polyamine antiporter, APA family, K03294) and cspA (cold shock protein, K03704) were significantly decreased. In the Tsi group, the abundances of ABC.PA.P (polar amino acid transport system permease protein, K02029), GST (glutathione S-transferase [EC: 2.5.1.18], K00799), ABC.PA.S (polar amino acid transport system substrate-binding protein, K02030), and lacI (LacI family transcriptional regulator, K02529) were significantly increased, while ABC.CD.A (putative ABC transport system ATP-binding protein, K02003) and ABC.CD.P (putative ABC transport system permease protein, K02004) were significantly decreased (*p* < 0.05).

In the Pathways database, compared with the control group, the Ppl group exhibited significant shifts in the predicted abundance of metabolic pathways. The potential for the Calvin-Benson-Bassham cycle (CALVIN-PWY), the superpathway of L-serine and glycine biosynthesis I (SER-GLYSYN-PWY), gluconeogenesis I (GLUCONEO-PWY), and glycolysis I (GLYCOLYSIS) was significantly enhanced. In contrast, the potential for L-isoleucine biosynthesis I (PWY-5973), fatty acid elongation -- saturated (FASYN-ELONG-PWY), and fatty acid salvage (PWY-7094) was significantly reduced. In the Ceq group, the potential for 5-aminoimidazole ribonucleotide biosynthesis II (PWY-6122) and the superpathway of 5-aminoimidazole ribonucleotide biosynthesis (PWY-6277) was significantly enhanced. In the Aca group, the potential for L-isoleucine biosynthesis IV (PWY-5104) was significantly increased, while the potential for urate biosynthesis/inosine 5′-phosphate degradation (PWY-5695) and pyruvate fermentation to isobutanol (PWY-7111) was significantly decreased. In the Tsi group, the potential for urate biosynthesis/inosine 5′-phosphate degradation (PWY-5695) was significantly increased, while the potential for the superpathway of adenosine nucleotides *de novo* biosynthesis I (PWY-7229) and the pentose phosphate pathway (non-oxidative branch, NONOXIPENT-PWY) was significantly decreased (*p* < 0.05).

### 3.6 Correlation analysis

**Figure 6:**
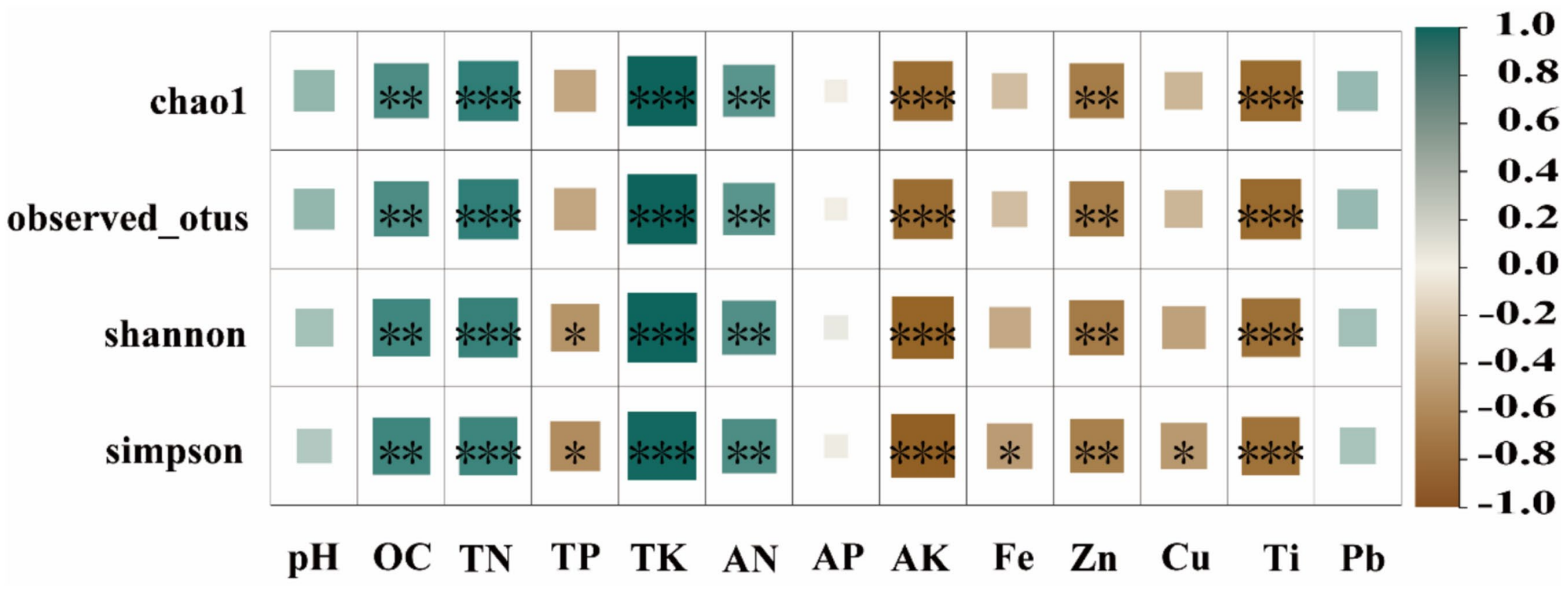
Correlation between soil physicochemical factors and *α*-diversity.

Pearson correlation analysis was employed to assess the correlation between soil physicochemical parameters and the *α*-diversity index of rhizosphere soil bacteria (Figure 6). The soil TK, OC, TN, and AN contents were found to be positively correlated with the α diversity index, except for dominance, according to the analysis results. In contrast, AK, Ti, and Zn contents were significantly negatively correlated with α-diversity indices (except for dominance). The TP content was negatively correlated with the Pielou_e, Simpson, and Shannon indices and positively correlated with dominance (*p* < 0.05).

**Figure 7:**
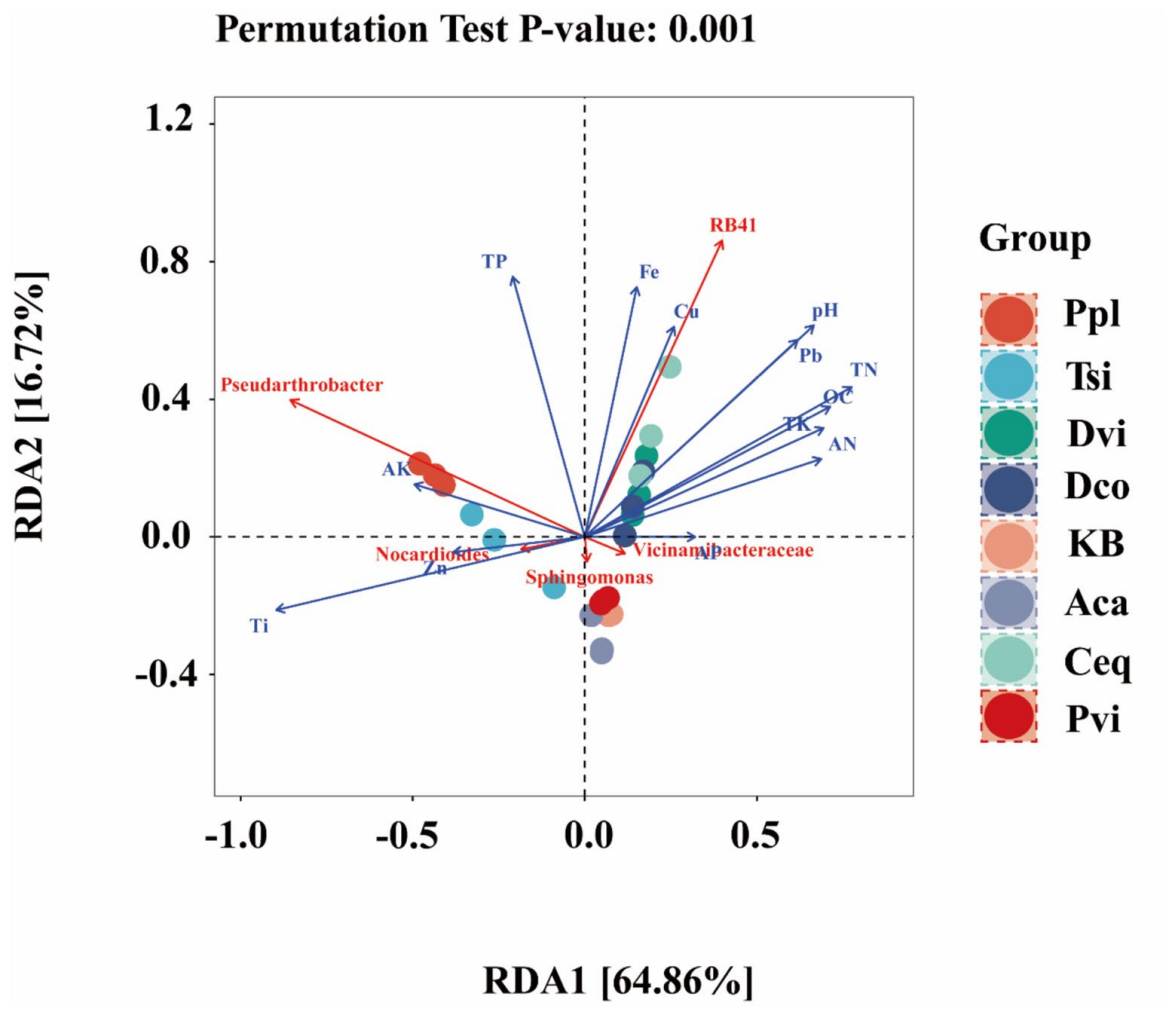
Redundancy analysis (RDA).

To further elucidate the specific effects of environmental factors on the rhizosphere soil and the endophytic bacteria in the rhizosphere, we performed redundancy analysis, focusing on the top 10 most abundant microbial species at the genus level and their relationship with soil environmental factors (Figure 7). RDA1 and RDA2 could explain 66.86 and 16.72%, respectively, which significantly revealed the complex relationship between environmental factors and the composition of microflora (*p* < 0.001). *Pseudarthrobater* showed a positive correlation with AK, while *Nocardioides* exhibited a positive correlation with Ti and Zn contents (*p* < 0.001). *RB41* was significantly positively correlated with Cu and Fe contents (*p* < 0.001).

## 4 Discussion

### 4.1 Impact of mining activities on soil physicochemical properties

The structure and diversity of plant rhizosphere bacterial communities are primarily influenced by soil physicochemical properties (Luo et al., 2025). Soil degradation caused by mining activities primarily manifests as soil structure disruption and nutrient depletion (Liu et al., 2024). pH can directly influence heavy metal behavior, nutrient availability, and microbial community composition (Xiao et al., 2021). In this study, soils in the mining area generally exhibited weak alkalinity, which may be related to the input of alkaline substances from mining dust and the formation of iron oxides (such as Fe(OH)₃) under oxidizing conditions (Huang et al., 2019). As the core of soil fertility and microbial activity, organic matter drives nutrient cycling in rhizosphere interactions (Liyang et al., 2024). OC levels in the experimental group were consistently lower than those in the control group, with the highest accumulation observed for Ceq. This may be because, under heavy metal stress, rhizosphere microorganisms accelerate the decomposition of organic matter to meet high energy demands in order to sustain basic life activities.

Nitrogen, phosphorus, and potassium form the foundation of soil fertility (Fan et al., 2021). Their total content indicators (TN, TP, TK) determine the potential for long-term plant growth, while the readily available components (AN, AP, AK) directly regulate the immediate nutritional status of plants (Pei et al., 2020). In terms of TN content, the rhizosphere concentrations of Ceq, Dco, and Dvi were significantly higher than those of KB. This may be attributed to the biological nitrogen fixation of Ceq (Liu Qiang, 2002) and the enrichment of rhizosphere nitrogen-fixing microorganisms in the latter two. Furthermore, the AN content in all plants was below the KB level. This may indicate that under specific conditions in the mining area, the demand intensity for AN in the rhizosphere microenvironment far exceeds its supply capacity. Soil phosphorus reserves serve as a vital source for plant growth and development (Billah et al., 2019). The TP content of all six plant species except Pvi exceeded KB, while the AP content of all seven plant species fell below KB. This may be because although plant-microbe systems can activate insoluble phosphorus pools in soil, the activated phosphorus is rapidly absorbed or sequestered. In contrast, AK in the rhizosphere of all plant species in this experiment exceeded KB. This suggests that potassium activation efficiency is relatively high and may not be the primary limiting factor in this system. Meanwhile, compared to the control area (KB), we observed a significant enrichment of iron (Fe) and copper (Cu) in the plant rhizosphere. Heavy metals such as iron and copper can disrupt the integrity of microbial cell membranes, interfere with enzyme function, and induce oxidative stress (Jia-wen et al., 2023; Yongli et al., 2014; Yunchuan et al., 2025). The combined effects of heavy metal toxicity and nutritional limitation act as the primary drivers of microbial diversity loss and community restructuring (Wei et al., 2024; Jing et al., 2024).

In summary, mining activities profoundly impact nutrient cycling and the rhizosphere microbiome by altering soil physicochemical properties. Previous studies have demonstrated the potential of Pvi, Ceq, and Dvi in managing heavy metal pollution. This research further supports their high applicability in ecological restoration within mining areas.

### 4.2 Microbial community restructuring

The composition and diversity of plant rhizosphere microbial communities are significantly regulated by soil environmental factors (Naz et al., 2022; Zeng et al., 2024). This study further confirms that iron ore mining activities significantly impact the alpha diversity of rhizosphere bacterial communities by altering soil physicochemical properties and plant-microbe interaction patterns (Qiu et al., 2024). Analysis of rhizosphere soils from different plant species revealed that the chao1 and observed-otus indices for Dco, Ceq, and Pvi were higher than those for KB, while the remaining four plant species showed lower values than KB. This may be attributed to the fact that different plants shape distinct rhizosphere microenvironments through their specific root exudates and ecological functions, thereby regulating microbial community composition. Correlation analysis further revealed that *α*-diversity indices showed significant negative correlations with AK, Ti, and Zn concentrations, while exhibiting positive correlations with TN and OC concentrations. This suggests that organic carbon may function as both a carbon source and a metal chelator, mitigating toxicity and promoting microbial proliferation. Conversely, elevated AK concentrations may lead to ion imbalance, while Ti and Zn exert direct toxic effects. These factors collectively contribute to reduced microbial diversity (Jenkins et al., 2020; Huang et al., 2023).

Bacterial communities play a crucial role in plant–soil interactions. This study reveals that under iron stress conditions, the rhizosphere microbial communities of seven plant species are dominated by Actinobacteria and Proteobacteria. This finding is consistent with previous research (Xu et al., 2021), which indicates that increased iron content in the plant rhizosphere promotes the enrichment of actinomycetes. The cell walls of *Actinomycetes* contain components such as bacitracin and peptidoglycan, whose surface functional groups can adsorb heavy metal ions through ion exchange (Rizvi and Saghir Khan, 2019; Yang et al., 2024), which may be the reason why Actinobacteria became the dominant phylum. On the other hand, the slime molds exhibit high metabolic plasticity, enabling them to efficiently utilize root exudates (Li Z. et al., 2025). This makes them crucial microbial partners for plants under nutrient-deficient and heavy metal-stressed conditions.

At the species level, the seven plant species exhibited distinct patterns of rhizosphere microbial recruitment. The rhizosphere of Ppl significantly enriches *Pseudarthrobacter* from the Actinobacteria phylum. This genus can secrete plant growth hormones to directly promote plant growth (Patel and Saraf, 2017). It is speculated that it may also enhance plant colonization capacity under stress conditions by secreting iron carriers to chelate iron ions with organic acids and dissolving immobilized heavy metals. The rhizosphere of Ceq, Dco, and Dvi primarily harbors the *RB41* genus from the Acidobacteria phylum. This oligotrophic microbial community (Stone et al., 2021) adapts to low phosphorus stress by hydrolyzing oligosaccharides and plays a crucial role in carbon, nitrogen, and phosphorus cycling (Zeng et al., 2022). This study further supports the notion of their significant ecological functions in stressed soils. The rhizosphere of Aca is primarily enriched with *Ellin6067* from the Verrucomycota phylum and *KD4-96* from the Bacillales phylum. The former phylum possesses a large genome and diverse metabolic potential (Narsing Rao et al., 2022), potentially conferring antioxidant and extracellular polymer secretion capabilities. While certain groups within the latter phylum possess anaerobic photosynthetic potential (Ye et al., 2024), potentially providing energy supplementation to the rhizosphere microenvironment. Together, these microorganisms assist plants in coping with oxidative stress and nutrient imbalance induced by iron stress.

Functional prediction analysis based on PICRUSt2 indicates that root-associated bacterial communities in different plants exhibit distinct metabolic reprogramming. The glycolysis and serine/glycine biosynthesis pathways in Ppl rhizosphere microorganisms showed significantly upregulated potential, indicating enhanced energy metabolism demands and potentially promoting antioxidant synthesis (Dao et al., 2025). Ceq root zones exhibited increased potential for DNA methyltransferase expression, suggesting that epigenetic regulation may be involved in their stress response (Xu et al., 2025). Correspondingly, the expression of glutathione transferase in the rhizosphere of Tsi plants was enhanced. As a key enzyme in glutathione conjugation reactions, this enzyme may alleviate heavy metal stress by strengthening the detoxification mechanism against exogenous toxins (Chhikara et al., 2024). Although the aforementioned metabolic potential requires further experimental validation, the results suggest that under identical environmental selection pressures, different plant species may evolve distinct ecological adaptation strategies by regulating the functional modules of rhizosphere microbial communities.

In summary, different plants exhibit distinct responses to adapt to their environments under identical stress conditions. Among these, Actinobacteria and Myxobacteria represent advantageous bacterial strains suitable for microbial inoculants, holding promise for future ecological remediation in heavy metal-contaminated environments.

## 5 Conclusion

This study investigated the effects of iron ore mining activities on rhizosphere bacterial communities of seven surrounding plant species. Results indicated that iron ore mining significantly increased TP, AK, and Fe content while decreasing AP, AN, and OC content. It also impacted the richness and diversity of rhizosphere bacterial communities. Using *α*-diversity indices, we found that Dco, Ceq, and Pvi increased the richness of rhizosphere bacterial communities, while *β*-diversity indices indicated significant differences in bacterial community structure among different plant rhizosphere soils. Functional prediction revealed potential pathways for glycolysis and serine/glycine biosynthesis, with significantly up-regulated expression of glutathione transferases and DNA methyltransferases. Pearson correlation analysis revealed significant negative correlations between α-diversity indices and AK, Ti, and Zn content, while positive correlations were observed with TN and OC content. This study provides preliminary insights into the assembly mechanisms and functional adaptations of rhizosphere bacterial communities in different plant species within iron ore mining areas. The specific action mechanisms require validation through extensive experimental studies, which will offer important references for future ecological restoration of iron ore mines.

## Data Availability

The original contributions presented in the study are included in the article/supplementary material, further inquiries can be directed to the corresponding author.

## Footnotes

1
http://ccb.jhu.edu/software/FLASH/
